# The Directory of Second-Hand Booksellers and List of Public Libraries, British and Foreign

**Published:** 1889-09

**Authors:** 


*The Directory of Second-hand Booksellers and List of Public Libraries, British and Foreign.* Rochdale: James Clegg. 1888.

It was a capital idea to supply the book-hunter with a work on the lines of this one, and we shall doubtless in further issues see the book much improved. For instance, the information as to the special kinds of literature to be found with certain booksellers needs much amplification; and it would be a great comfort, upon going into a town for the first time, to know how access could be obtained to many of the libraries mentioned at pp. 59—72. It is strange to see a list of "Bibliographical Works of Reference" which contains no mention of Herbert's edition of Ames's *Typographical Antiquities*, of the British Museum *Catalogue of Early Printed Books*, or of Mr. Arber's *Transcript of the Stationers' Registers*. On the other hand, the list of "Journals of the Book Trades" is widely comprehensive; and some of the higher class literary papers must be astonished at finding themselves among much excellent but merely commercial company. It would be interesting and certainly appropriate to have in future editions a chapter on the history of bookselling.

We hope it is intended to make this publication an annual one.

